# mCOPA: analysis of heterogeneous features in cancer expression data

**DOI:** 10.1186/2043-9113-2-22

**Published:** 2012-12-10

**Authors:** Chenwei Wang, Alperen Taciroglu, Stefan R Maetschke, Colleen C Nelson, Mark A Ragan, Melissa J Davis

**Affiliations:** 1 Institute for Molecular Bioscience, The University of Queensland, Brisbane, 4072, Australia; 2 Australian Prostate Cancer Research Centre – Queensland, Queensland University of Technology, Brisbane, 4102, Australia

**Keywords:** Cancer, Outliers, Expression data, Expression profile, Cluster, Subtype, Heterogeneous, Bioinformatics, Percentile, Feature selection

## Abstract

**Background:**

Cancer outlier profile analysis (COPA) has proven to be an effective approach to analyzing cancer expression data, leading to the discovery of the TMPRSS2 and ETS family gene fusion events in prostate cancer. However, the original COPA algorithm did not identify down-regulated outliers, and the currently available R package implementing the method is similarly restricted to the analysis of over-expressed outliers. Here we present a modified outlier detection method, mCOPA, which contains refinements to the outlier-detection algorithm, identifies both over- and under-expressed outliers, is freely available, and can be applied to any expression dataset.

**Results:**

We compare our method to other feature-selection approaches, and demonstrate that mCOPA frequently selects more-informative features than do differential expression or variance-based feature selection approaches, and is able to recover observed clinical subtypes more consistently. We demonstrate the application of mCOPA to prostate cancer expression data, and explore the use of outliers in clustering, pathway analysis, and the identification of tumour suppressors. We analyse the under-expressed outliers to identify known and novel prostate cancer tumour suppressor genes, validating these against data in Oncomine and the Cancer Gene Index. We also demonstrate how a combination of outlier analysis and pathway analysis can identify molecular mechanisms disrupted in individual tumours.

**Conclusions:**

We demonstrate that mCOPA offers advantages, compared to differential expression or variance, in selecting outlier features, and that the features so selected are better able to assign samples to clinically annotated subtypes. Further, we show that the biology explored by outlier analysis differs from that uncovered in differential expression or variance analysis. mCOPA is an important new tool for the exploration of cancer datasets and the discovery of new cancer subtypes, and can be combined with pathway and functional analysis approaches to discover mechanisms underpinning heterogeneity in cancers.

## Background

Within a type of cancer, tumours are frequently heterogeneous at the molecular level. Some of this diversity may describe cancer subtypes, but even within a subtype, individual primary and metastatic lesions often differ from one another. Modern microarrays can measure the expression of >10^5^ protein coding or noncoding features, thereby capturing an important dimension of this diversity. Until recently, statistical analysis of expression-microarray data typically focused on the recognition of molecular subtypes and discovery of characteristic biomarkers; instances of within-subtype heterogeneity (outliers) were either removed from the analysis, or more rarely explored as a source of information on rare events. Cancer outlier profile analysis (COPA) was developed to identify transcripts up-regulated in only a small subset of cancer samples [1], and has successfully been used to identify recurrent TMPRSS2 gene fusions in prostate cancer.

The original outlier detection method implemented a data transformation in which the median of each expression feature across all samples is scaled to 0.0 and the mean absolute deviation to 1.0. Features are then ranked based on their value at the 75th, 90th or 95th percentiles. The COPA method has been integrated into Oncomine [2], but its use is limited to Oncomine datasets. The only independent implementation of COPA is available as a package in R [3]. This implementation assigns outliers by applying a flat threshold to the COPA score of all features, instead of looking at the range of expression of each individual feature to determine a feature-specific threshold. Genes are grouped into mutually exclusive gene pairs, and ranked according to the number of tumour samples in which either of the genes is an outlier. This R implementation was developed to identify expressed oncogenic gene fusions according to the original publication of the method [1], but in practice fails to capture the full complexity and sensitivity of that analytical approach, instead providing a very circumscribed implementation for detection of oncogenic gene fusions.

Within our research program on cancer networks [4-6] we identified the need for a more-flexible extension of outlier profile analysis that supports (i) the independent analysis of our own microarray data, without limitation to datasets available through Oncomine; (ii) sensitive feature-specific threshold selection (more in line with the original COPA method) to account for variation in feature expression; (iii) the generation of outlier profiles for custom analysis for which the mutually exclusive gene pairs output of the COPA R package is not suitable; and (iv) the identification of outlier profiles not only for over-expressed features, but for under-expressed features as well. Beyond these, considerable potential exists for extending outlier profile analyses more broadly to further types of use.

Here we present a modified COPA analysis program, mCOPA, which incorporates statistical refinements to outlier detection, including improvements in the calculation of percentiles [7,8] and thresholds for outlier identification [9-11]. Additionally, we identify under-expressed outlier genes, a category identified in neither the original method nor the later R package.

To assess the performance of mCOPA as a feature-selection algorithm applicable to microarray datasets, we evaluated the utility of features selected by mCOPA in separating clinically defined cancer subtypes represented in expression microarray data. We evaluated the quality of features selected by mCOPA and by three other algorithms (COPA, differential expression (DE) analysis, and variance analysis) on 12 publicly available datasets. Quality was assessed based on the ability of feature sets to cluster samples into recognised subtypes. As it has been shown that clustering performance varies greatly depending on the dataset and the clustering method [9], we evaluated four different clustering methods: K-means [12], PAM [13], clues with CH strength index, and clues with the Silhouette (Sil) strength [14].

We also perform a detailed analysis of one of these 12 datasets (the dataset of Tomlins *et al.*[15]), demonstrating the application of mCOPA in conjunction with pathway mapping and functional analysis. The results of our comparison and detailed analyses provide guidelines for the efficient use mCOPA, and highlight novel ways in which this approach can be applied to analyse and interpret microarray data. The mCOPA software is freely available from www.bioinformatics.org.au/mCOPA.

## Methods

### Data format and algorithm

mCOPA takes as input a matrix of preprocessed microarray data, with rows representing features and columns containing sample data. The first columns contain data from the normal samples, followed by the tumour samples. Example input and output files are included with the application, and a flowchart of the workflow is provided in the user manual. The COPA transformation [1] is the first step of the workflow. Using the transformed COPA scores, the 25th, 75th and the user specified upper and lower percentile values (for example, but not limited to 90th or 95th and 5th or 10th percentiles) of features are calculated separately for tumour samples and normal samples.

We define over-expressed outliers as features that have a COPA-transformed value greater than the 75th percentile plus 1.5 times the inter-quartile value (calculated from the tumour samples). Under-expressed outliers are defined as features with a COPA-transformed value less than the 25th percentile minus 1.5 times the inter-quartile value (calculated from the tumour samples) [16]. This procedure can result in outliers in both normal and tumour samples, so we apply the following criteria to filter the initial outlier set in order to (i) maximise the difference between normal and tumour profiles, and (ii) remove any outliers that occur in normal samples:

1. We require the feature to be an outlier in at least one tumour sample;

2. We require that the feature is not an outlier of the same direction (up or down) in any normal samples (*i.e.* for up-regulated outlier features, it cannot be an up-regulated outlier in any of the normal samples, but it can be a down-regulated outlier in normal samples); and

3. We require the log2 of the absolute value of the fold change between the nominated percentile values of tumour samples and normal samples to be larger than 2.

In theory the outlier analysis and subsequent filtering does not prevent probes from being detected in different samples as over- and under- expressed outliers, however in practice we rarely observe this to occur, and no outliers are detected in both directions in the Tomlins data we analyse in detail below.

### Evaluation of feature selection

To evaluate the ability of features selected by mCOPA to cluster clinically defined cancer subtypes, and compare the results with other feature selection methods, 12 cancer datasets [15,17-27] (see Additional file 1 – Public datasets for details and accession numbers of these datasets) were downloaded from ArrayExpress [28] or GEO [29] when not available from the former. Subtypes for cancer samples in each dataset were defined by clinical annotation. Cancer subtypes with three or fewer samples were removed from the datasets.

Normalisation was verified by box-plot (to determine if data were median-centered). Non-normalised datasets were converted to ExpressionSet objects using Biobase. We applied a log2 transform and then normalised using the *normalize* function of the Affy Package in R. [30]. Finally, we examined the distribution of every dataset to ensure all data were appropriately and consistently normalised. DE analysis was performed using the Limma R package [31] with an adjusted p-value threshold of 0.01 for selecting features. For mCOPA, the 90th and 10th percentile values were used for selecting over- or under-expressed outlier features. Over- and under-expressed outlier outputs of mCOPA were combined into a single feature list to represent mCOPA outliers similar to DE analysis output, which contains both over- and under-expressed outputs. COPA outputs were ordered according to 90th percentile values, and the top-ranked features (constituting a set of over-expressed outliers) were selected to give a feature set equivalent in size to the mCOPA output. The variance method of feature selection ranks features according to their variance in expression; 1000 features with the largest variances were selected for this evaluation.

The features selected by the four methods were then passed to four clustering methods: K-means (KM), PAM, and Clues with the CH strength index or the Silhouette (Sil) strength index. The Stats package from R was used for K-means clustering, with the settings of 20 repeats and “MacQueen” algorithm. The Cluster package [32] of R was used for PAM clustering method with default settings. The Clues package [14] of R was used with the CH or Sil estimators for cluster numbers and with the default settings. For K-means and PAM, the number of clusters was specified, whereas Clues automatically determines the number of clusters using the CH or Sil strength index.

The quality of the clustering was measured by the Adjusted Rand Index (ARI) [33]. The ARI corrects for bias that might occur in clusters due to chance, given the relative sizes of subtype groups. ARI falls between “1” and “-1”; ARI = 1 indicates a perfect clustering, and ARI = 0 a clustering no better than chance. ARI can be smaller than zero, indicating an anti-correlation, thus low-quality clustering result. Given a dataset of n samples S = [X1, … , Xn] with the two partitions K = [K1,…,Kd] and L = [L,…,Lc], the adjusted Rand index is computed as:

(1) $$\mathrm{ARI}\;=\frac{\sum_{\mathrm{ij}}\left(\begin{array}{l} n_{\mathrm{ij}}\\ 2 \end{array}\right)-\left[\sum_{i}\left(\begin{array}{l} n_{i}\\ 2 \end{array}\right)\sum_{j}\left(\begin{array}{l} n_{j}\\ 2 \end{array}\right)\right]/\left(\frac{n}{2}\right)}{\frac{1}{2}\left[\sum_{i}\left(\begin{array}{l} n_{i}\\ 2 \end{array}\right)+\;\sum_{j}\left(\begin{array}{l} n_{j}\\ 2 \end{array}\right)\right]\;-\left[\sum_{i}\left(\begin{array}{l} n_{i}\\ 2 \end{array}\right)\;\sum_{j}\left(\begin{array}{l} n_{j}\\ 2 \end{array}\right)\right]/\left(\frac{n}{2}\right)}\;=\frac{\mathrm{Index}-\mathrm{Expected}\;\mathrm{Index}}{\mathrm{Maximum}\;\mathrm{Index}-\mathrm{Expected}\;\mathrm{Index}}$$

where ∑_ij_ n_ij_ represents the number of sample pairs in the same cluster in K and in the same cluster in L. ∑_i_ n_i_ represents the number of samples in each cluster in K, and ∑_j_ n_j_ the number of samples in each cluster in L. Each of the four feature selection methods was combined with each of the four clustering methods, resulting in 16 ARI scores for each of the 12 data sets. Since normal distribution of these data cannot be assumed, we applied the parameter-free Kruskal-Wallis test [34] to identify statistically significant differences in the ARI scores of the evaluated methods.

The *Mclust* package [35] for R was used to generate ARI scores from partitions of clustering methods. The *pgirmess* package [36] of R was used for the Kruskal-Wallis test to compare the ARI scores obtained in the previous step, using a threshold of p<0.05.

### Analysis of the Tomlins *et al.* prostate cancer dataset

mCOPA was applied to the dataset of Tomlins *et al.* 2007 [15] to generate lists of over- and under-expressed outliers. The processed prostate cancer dataset [15] was downloaded from ArrayExpress (id: E-GEOD-3325). Probe IDs (first column) and expression values (last columns) were extracted from each sample file and written into a matrix format. Only normal epithelial, tumor-adjacent normal, pre-invasive neoplasm (PIN), prostate cancer (PCA) and metastatic (MET) samples were included in this analysis. Probes and samples with more than 40% missing values were excluded from this analysis. Any remaining missing values were imputed using the *ImputeMissingValuesKNN* module from GenePattern, with k set to 10.

A matrix file with 16572 rows and 87 columns was used in the analysis (DemoInput.txt available from the mCOPA website [37]). The microarray annotation file was downloaded from GEO (access number: GPL2013). The *toptable* command of the *Limma* package in R was used for output analysis results. An adjusted p-value of 0.01 was set as the threshold. All probes in the *toptable* output file with an adjusted p-value of less than 0.01 were chosen as DE probes, and the corresponding expression data were then extracted and used as input for the following clustering analysis. 90th and 10th percentile values were used in the mCOPA up- and down-regulated outlier selection, and columns of the output lists containing the expression values of up- and down-regulated outlier probe lists were extracted and merged into a single file for the following clustering step. The *HierarchicalClustering* module of the GenePattern package [38] was used for clustering. Pearson correlation was chosen as the column and row similarity measure, and pairwise average linkage was used as the linkage method; no log transformation, row centering, row normalisation, column centering or column normalisation was performed.

Following the clustering of the samples based on the selected features, the metastatic cluster was selected and separate up- and down-regulated outlier feature lists were generated using the getSubtypeProbes.pl function of the mCOPA package. These two feature lists were then converted into gene lists based on the array annotation file. Outlier profiles for the samples from the metastatic subtype were then used to generate sample-specific outlier lists. Pathway analysis in the software package MetaCore from GeneGo Inc. was then used to analyse these outlier lists.

## Results and discussion

### Implementation

Our implementation of mCOPA is a set of Perl scripts available at http://www.bioinformatics.org.au/mCOPA. The mCOPA method takes normalised expression values in matrix format as input, with each line representing a feature, each column representing a sample, and normal samples followed by tumor samples. The user also needs to indicate the number of normal samples (*n*) in the dataset (present as the first *n* columns of samples), and nominate the percentile values used for up- and down-regulated outlier detection (see Methods, and user documentation available on the mCOPA website [37]. If the experiment has no normal samples, the first *n* samples provided in the file will be treated as the control set for the experiment, and used in the place of normal samples. Two output files are produced, one for up- and one for down-regulated outlier features. Details on use of this application and example data are available online.

mCOPA has been designed to select outliers as features that may be used in subsequent downstream analysis (as demonstrated in the analysis of the Tomlins *et al*. (2007) dataset), functionality absent from the earlier R implementation, which outputs only mutually exclusive outlier pairs. Additionally, the earlier R implementation of COPA does not use established definitions of outliers (*i.e*., based on the feature specific distribution of values). Instead, it applies a hard-coded threshold which calls any feature an outlier in samples in which its COPA score exceeds 5, regardless of the distribution of these scores across all the samples, whereas our method applies sensitive, feature specific criteria to determine if features are outliers in a given sample. The COPA implementation in the Oncomine database only ranks genes, with no threshold applied to clearly define which genes are outliers. The additional restriction that it can only be applied to datasets within Oncomine reduces its use as a general analytical tool.

The mCOPA package can be applied to any given expression dataset in which two conditions are defined, although here we discuss its application to cancer expression data containing normal and cancer samples as the two conditions. In cases where there are no normal samples available, a subset of disease samples could be substituted for the normal samples and used as a control set to contrast with the samples of interest. For example, mCOPA could be used to identify outliers in aggressive tumours but not indolent tumours, or in high-grade tumours but not low-grade tumours.

In addition to lists of outlier features, mCOPA provides outlier profiles: strings composed of 1, -1 or 0, indicating the samples in which a given feature is either an over-expressed outlier, under-expressed outlier or non-outlier. Further, there is a function getSubtypeProbes.pl in the mCOPA package, which can identify which features are outliers only in a given set of samples. This function is useful in studying a subtype of cancers once clinical data are integrated or the samples are clustered.

### Feature selection: clustering

**Figure 1:**
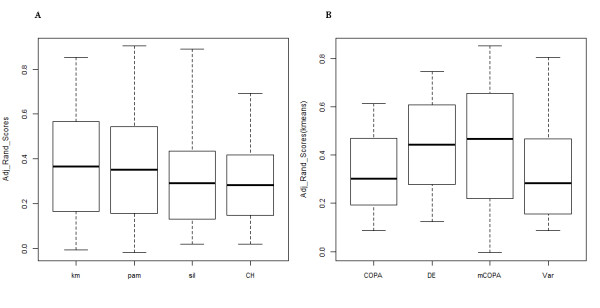
**Statistical evaluation of ARI scores.** (**A**) Boxplots of ARI for four clustering methods across all 12 datasets. K-means achieves the highest ARI; (**B**) Boxplots of ARI for four feature selection methods based on k-means clustering results across 12 datasets. mCOPA achieves the highest ARI.

We systematically evaluated the performance of features selected by mCOPA relative to those selected by DE, variance (*i.e*. selecting the most-variable probes [39,40]) or the original COPA algorithm in the task of clustering cancer subtypes. We selected 12 expression datasets for which cancer subtypes had been determined based on clinical annotation, not by molecular profile (see Methods Section and Additional file 1). To minimise the possibility of certain clustering methods favoring particular feature selection approaches, we evaluated clustering performance of the different feature sets using four clustering approaches. Clustering quality scores (ARI values) for the 12 datasets are presented in Table 1. The mCOPA method achieved the highest score for 7 out of the 12 datasets, consistently providing more-accurate clustering performance. None of the four clustering algorithms achieved significantly better performance than the others (Kruskal-Wallis test), although K-means tended to perform slightly better than the others (Figure 1A). We therefore compared ARI scores of the four feature-selection approaches using only K-means for clustering, in order to evaluate the feature-selection algorithms. Combined with K-means, mCOPA was the best-performing feature selection algorithm (Figure 1B) although the Kruskal-Wallis test did not find the distribution of ARI scores to be significant at the acceptance threshold.

**Table 1:** ARIs of four feature selection methods combined with four clustering methods across 12 datasets

**Feature selection + clustering method**	Datasets* (Details presented in Additional file**1****– Public datasets)**											
	**Pr**	**C**	**Mn**	**R1**	**R2**	**NPh**	**Lm**	**R3**	**B**	**T**	**Br**	**L**
COPA+CH	0.12	0.04	0.69	0.20	0.64	0.15	0.23	0.38	0.06	0.05	0.16	0.45
COPA+KM	0.30	0.16	0.53	0.62	0.33	0.31	0.25	0.54	0.09	0.23	0.12	0.41
COPA+PAM	0.13	***0.18***	0.60	***0.90***	***0.81***	0.36	0.26	0.57	−0.02	0.31	0.12	0.35
COPA+SIL	0.04	0.08	0.69	0.20	0.30	0.15	0.33	0.43	0.06	0.05	0.07	0.55
DE+CH	0.17	0.15	0.21	0.24	0.53	0.36	0.38	0.28	0.21	0.52	0.12	0.44
DE+KM	0.29	0.15	0.51	0.75	0.59	0.34	0.38	0.65	***0.27***	***0.63***	0.12	0.54
DE+PAM	0.35	0.13	0.24	0.79	0.76	0.34	0.26	0.56	0.11	0.46	0.16	0.43
DE+SIL	0.17	0.15	0.33	0.24	0.53	0.14	0.38	0.28	0.21	0.52	0.12	0.44
mCOPA+CH	0.29	0.15	0.60	0.55	0.40	0.35	0.38	0.39	0.06	0.52	0.11	0.30
mCOPA+KM	0.46	0.01	0.79	0.68	0.48	0.36	***0.45***	***0.85***	0.00	***0.63***	0.08	***0.62***
mCOPA+PAM	***0.47***	0.10	0.55	0.82	0.54	***0.45***	0.44	0.49	0.03	0.50	***0.20***	0.44
mCOPA+SIL	0.29	0.14	0.60	0.61	0.40	0.35	0.38	0.39	0.06	0.52	0.11	0.30
VAR+CH	0.14	0.08	0.69	0.32	0.34	0.28	0.38	0.57	0.02	0.26	0.13	0.45
VAR+KM	0.16	0.16	0.47	0.81	0.47	0.23	0.34	0.64	0.09	0.15	0.17	0.41
VAR+PAM	0.17	0.16	***0.88***	0.81	0.43	0.02	0.26	0.61	0.10	0.06	0.15	0.33
VAR+SIL	0.14	0.08	0.69	0.89	0.34	0.28	0.38	0.57	0.02	0.12	0.13	0.59

Note: the ARI scores in italicized bold indicate the best performing method for each dataset. Datasets in bold indicate those in which mCOPA provided the most informative feature selection for the clustering of clinical subtypes.

*Datasets: Pr (Prostate: GSE6099); C (Cervical: GSE7410); Mn (Melanoma: GSE7553); R1 (Renal: GSE11024); R2 (Renal: GSE11151); NPh (Nasopharangeal: GSE12452); Lm (Lymphoma: GSE12453); R3 (Renal: GSE15641); B (Brain: GSE15824); T (Thyroid: GSE29265); Br (Breast: GSE29431); L (Lung: GSE32036).

While the ARI provides an objective index for comparing clusters with known clinical groups, there are some problems with this approach. Some datasets do not cluster well and score very low values; in particular, GSE7410 (Cervical), GSE15824 (Brain) and GSE29431 (Breast) have very low ARI scores and the highest score for these datasets is lower than the lowest score across the many of the other datasets (see Additional file 2). We analysed each experiment separately to determine if feature-selection approaches showed significantly different ARI scores in experiments where they achieved the best performance (Additional file 3). mCOPA produced significantly different results in five of the experiments, and in all but one of these cases, it was the top-performing method. Likewise, DE was the best performer in two of the three experiments in which it produced significantly different ARI scores. For the other two methods, significance was associated with poor performance only. What this analysis highlights is that datasets are highly variable in terms of the accuracy with which they can be clustered, and no one method works the best in all cases. However, mCOPA consistently selects features that support the most-accurate clustering, making it an attractive feature selection approach for clustering samples.

### Feature selection: different features and different biology

Features selected by the four approaches are usually distinctly different (Additional file 4). We examined overlap between features selected by mCOPA, DE and variance approaches, and found little overlap: on average, two-thirds of the features selected by mCOPA are unique to that method, although the proportion of overlapping features varies depending on the number of differentially expressed features with p-values smaller than 0.01.

We next asked whether the different feature sets correspond to unique biology. After mapping features to GO terms using DAVID [41,42], we observe a similar trend: features selected by mCOPA map to GO terms of which 62% on average are unique to the mCOPA feature set, and are not enriched in the DE- or variance-selected feature sets (Additional file 5). This semantic analysis demonstrates that mCOPA unveils a different kind of biological functionality than is found by DE or variance. Typically, fewer ontology terms map to mCOPA features, and thus capture more-focused functions. In those cases for which mCOPA exhibits a low degree of functional uniqueness, all feature selection methods show the same lack of unique biology, *i.e.* in some datasets all feature-selection approaches converge on a consistent biological signature. Interestingly, these are the datasets with the most mapped functions, not the fewest. Where fewer GO terms are associated with feature sets, the individual methods tend to show distinctly different biological properties. Given that mCOPA focuses on outliers present in only a few samples, we propose that the exploration of such heterogeneity contributes to the ability of feature sets selected by our method to distinguish cancer subtypes, and demonstrate how outlier analysis achieves such specificity in a detailed analysis of prostate cancer data (below).

### Application: Tomlins *et al.* 2007

To explore the performance of mCOPA in more detail, we applied our method to the prostate cancer dataset of Tomlins *et al.*[15]. We examine the ability of outlier features to cluster the subtypes present in this dataset, explore outliers that are unique to one of the resulting clusters (corresponding to metastatic tumours), analyse sample-specific outliers, and integrate semantic analysis to identify genes that are potential novel tumour suppressors in prostate cancer.

**Figure 2:**
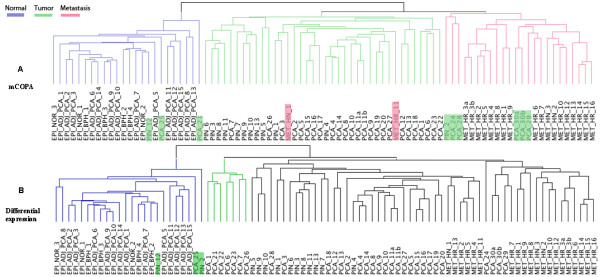
**The clustering result comparison for mCOPA and differential expression (DE) analysis.** (**A**) mCOPA produces three clusters: normal samples (blue), tumour samples (green), and metastasis samples (red). (**B**) DE also produces three clusters: normal samples (blue), a small cluster of tumour samples (green) and a large cluster of mixed tumour and metastatic samples. Misclassified samples are highlighted.

Both the mCOPA and DE feature sets (see Additional file 6) clearly separate the normal samples (blue) (Figure 2). mCOPA, however, separates the metastatic samples (red cluster, Figure 2A) from other pathological samples (green cluster, Figure 2A), whereas the DE genes put all these subtypes into one large cluster (black cluster, Figure 2B).

As we had previously observed that feature sets corresponded to genes with different functions or that participate in different processes, we performed DAVID functional analysis of differentially expressed and outlier genes from the Tomlins *et al.* data [15] in order to compare and contrast the insights provided by these two approaches. The most significant functional clusters for under-expressed outliers involved apoptotic signaling and signal transduction, and regulation of cell adhesion. Significant functional clusters of down-regulated DE genes involved vesicle and membrane proteins, and oxidative metabolism. Over-expressed outliers were characterised by clusters involving mitotic cell cycle and protein complexes, while up-regulated DE genes had clusters involving cadherin signaling and the cytoskeleton. The different biology revealed by DE and outlier features in this analysis are consistent with differences in Gene Ontology analysis observed in our more general analysis of expression datasets (above).

### Pathway analysis of outliers in the metastatic cluster

**Figure 3:**
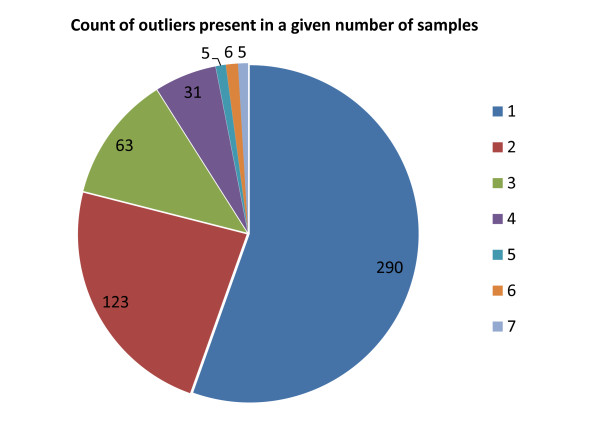
**Analysis of the number of samples sharing a given outlier.** Most outlier features are outliers in only a small number of metastatic samples, with very few outliers shared across more than three samples. Very similar proportions are observed when sample counts for either under- or over-expressed outliers are considered separately. Counts are only shown for those outliers that occur in the metastasis cluster. A further 201 outliers map exclusively to non-metastatic samples.

Outlier analysis lends itself to a different kind of pathway analysis than traditionally applied to differentially expressed gene sets. Whereas pathway analysis of a set of differentially expressed genes can provide insight into mechanisms that are disrupted generally across tumour samples, outliers are, by definition, disrupted only in a very small number of tumours (see Figure 3). This means that the traditional application and interpretation of pathway enrichment results is not appropriate for outlier sets. Instead, outlier lists can be used to infer mechanisms that are specifically disrupted in single tumour samples, or in small sets of samples. We suggest two approaches:

1. Outlier lists for subtypes can be used to perform pathway enrichment, identifying pathways disrupted by outliers in that subtype; outliers in each pathway can then be mapped back to the samples in which they occur to determine if (i) pathway disruption is general within the subtype (*i.e.* many samples within the subtype contribute different outliers to the pathway, implying that different molecular mechanisms nonetheless result in disruptions to the same pathway), or (ii) pathway disruption is specific to one or more samples within the subtype (*ie*. all the outlier genes mapped to a specific pathway come from a small number of samples within the subtype).

2. Outlier lists for each sample in an experiment can be extracted from the outlier profiles generated by mCOPA. These lists of over- and under-expressed outliers can then be used to identify significant pathways disrupted in each sample. Pathways can then be compared across samples to determine if samples converge on a common pathway, or contain specific and unique pathway disruptions.

Following the first approach, we analysed pathway enrichment in the set of outliers associated with the metastatic prostate cancer cluster (red cluster, Figure 2). The two most significantly expressed pathways were EGFR signaling (p-value 4.55e-7), and PTEN signaling (p-value 2.49e-5). The EGFR signaling pathway contains six over-expressed outliers (EGFR, GRB2, PDPK1, PKC-theta, c-Myc, and FAK1), and one under-expressed outlier (ERBB2 also known as HER2). Each over-expressed outlier is found in one or two metastatic tumour samples, with only one tumour containing two over-expressed outliers in this pathway. Over expression of EGFR and components of its signaling pathway such as c-Myc are well known to be associated with metastasis [43-46]. Here, we are able to identify specific tumours with strong over-expression of different components of this pathway, indicating that through different genetic mechanisms, nearly half of our metastatic samples show hits to this pathway. Interestingly, only one metastatic tumour sample (MET_HR_10) shows strong loss of expression of ERBB2 (HER2), a gene often associated with promoting cell proliferation, particularly in breast cancer [47,48]. It is known, however, that loss of expression of ERBB2 is a feature of metastatic sites in breast cancer that is otherwise ERBB2 positive [49] and further that loss of ERBB2 expression has been strongly associated with progression to metastasis in osteosarcoma [50].

The PTEN signaling pathway presents another interesting case study for the use of outlier analysis, and contains five over-expressed outliers (EGFR, PDPK1, RHEB2, FAK1, GRB2). PTEN is a known tumour suppressor, and loss of PTEN function is associated with cancer progression [51,52]. PTEN normally inhibits integrin-mediated survival and migration [53]. Interestingly however, the outlier effect we observe in the PTEN pathway is not loss of the tumour suppressor, but very strong over-expression of a signaling factor downstream of integrin, FAK1 (PTK2), which is usually inhibited by PTEN [54]. While PTEN expression has not been lost, over-expression of its substrate has the potential to flood the inhibitory interaction between PTEN and FAK1, thus enabling the integrin signaling pathway to escape PTEN inhibition in two specific metastatic tumours. This observation, and the previous example, illustrates the power of combining outlier analysis and pathway analysis to identify heterogeneous disruptions within a cancer subtype. Such sample-specific observations will become increasingly valuable as clinical tools for molecular-targeted therapies in cancer treatment.

Following the second approach outlined above, an outlier list was extracted for each metastatic prostate cancer sample in the Tomlins dataset. Samples show high variability in the number of over- and under-expressed outliers they contain (ranging from 4 to 196 outliers). While 40% of all outlier features are present in only one metastatic tumour sample, fewer than 2% of the outliers are present in five or more samples (see Figure 3). Thus outliers represent features that reflect the unique molecular characteristics of tumours rather than general molecular characteristics.

**Figure 4:**
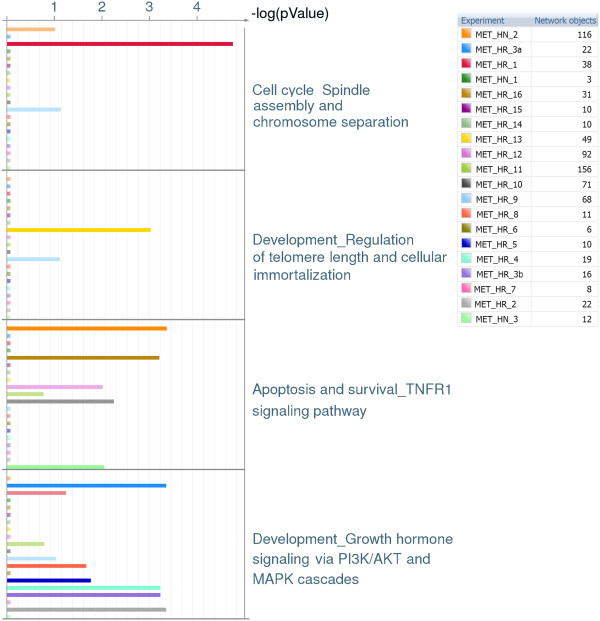
**Details of outlier pathway analysis highlighting the differences between pathways significantly disrupted in individual samples.** In the first two pathways, only a single sample shows significant pathway enrichment, whereas the last two pathways are more-generally affected in 15% and 30% of metastatic samples respectively.

Pathway enrichment analysis of the 20 outlier feature sets for the Metastatic tumours reveals an interesting pattern (see Additional file 7 and an extracted part of the supplementary figure shown in Figure 4). Many pathways, such as the first two shown in Figure 4 (Cell cycle spindle assembly, and Regulation of telomere length) are significantly enriched (i.e. p value < 0.01, thus –log(p value) >2) in the outliers of a single sample (here, MET_HR_1 and MET_HR_13 respectively), and very few pathways (such as Apoptosis and survival through TNF4 signaling and Growth hormone signaling via PI3K/ AKT) are disrupted more-generally (in this case, in three and six metastatic tumour samples respectively).

This alternative approach to pathway enrichment, where outliers from individual tumour samples are analysed instead of sets of broadly differentially expressed genes, generates highly specific insight into the biology of individual tumours, and demonstrates how our mCOPA method can be used to generate sample-, or patient-specific interpretations from high-throughput experimental results. Given that most cancer treatments work only for a subset of patients [55-57], approaches such as this provide important insights into the molecular differences that may underpin such differential response to treatment. Analysis of pathway disruption at the level of individual samples presents an important contribution to the development of more-personalised approaches to molecularly targeted therapeutics.

### Identification of tumour suppressors

The original COPA method was used in conjunction with a set of known oncogenes to identify up-regulated oncogenic outliers. Because our extension to the method identifies down-regulated outliers as well, we investigated whether mCOPA might identify tumour suppressors. A set of 727 cell cycle regulators containing many known and potential tumour suppressors was extracted from the Gene Ontology Database [58]. Of 223 down-regulated outliers, 12 genes are annotated cell cycle regulators. A search in the Cancer Gene Index database ( https://wiki.nci.nih.gov/display/cageneindex/) showed that four of these 12 genes are known tumour suppressors in prostate cancer (RBL2, CDK6, TP63, BIRC2), while five (SON, PAFAH1B1, PDCD4, RBBP8, DBC1) have been reported to be tumour suppressors in other cancers. The remaining three genes (FZR1, CDC14B, HEXIM1) represent potentially novel tumour suppressors.

We reviewed the annotation available for these three genes in Uniprot, and examined their expression profiles in Oncomine. The Fizzy-related protein homolog Fzr encoded by FZR1 plays a role in the degradation of positive regulators of cell cycle, and prevents entry into mitosis following DNA damage. TCGA datasets in Oncomine reveal that FZR1 has a significant loss of copy number in prostate, ovarian, lung, gastric, endometrial and breast cancers. Its expression is significantly reduced in 46 experiments. A similar trend is seen with the HEXIM1 gene, which encodes protein HEXIM1, a transcriptional regulator that acts as a general transcription inhibitor. This gene has significant copy number loss in prostate, ovarian, breast, colorectal and endometrial cancers, and is significantly under-expressed in 101 experiments collected in Oncomine. The third candidate tumour suppressor we uncovered, CDC14B, codes for the protein Dual-specificity protein phosphatase CDC14B, an essential regulator of the G2 DNA damage checkpoint. It does not show significant loss of copy number in TGCA prostate cancer data, but does show significant loss in breast, ovarian, renal, lung and endometrial cancers, and is significantly under-expressed in 84 experiments. Together these analyses demonstrate that these three genes are credible as potential tumour suppressors; they are subject to copy-number loss in a wide range of cancers, and are significantly under-expressed in a large number of microarray experiments.

## Conclusions

Here we have shown how mCOPA-derived cancer outlier profiles can be used to interpret cancer microarray data. We evaluated outlier profiles as a feature-selection method for clustering clinically defined cancer subtypes, and compared the performance of mCOPA to three other outlier selection approaches. mCOPA consistently selects features that are more informative. We hypothesise that this is because of the properties of outlier expression profiles, which capture the different molecular processes and networks disrupted in individual tumour samples. Approaches such as differential expression analysis, which identify features that are consistently different across cancer samples compared with normals, do not reveal this biological heterogeneity. Given the lack of overlap between genes and corresponding biology targeted by the feature selection methods we examined, we propose that researchers should explore multiple complimentary approaches, including mCOPA, in analysing high-throughput data, so as to exploit more fully the range of biology to which these approaches give privileged access.

Application of our method to the Tomlins *et al.* dataset [15] highlights the strength of our approach. We demonstrate the use of mCOPA to select features capable of accurately clustering cancer subtypes; we also show that these features represent distinct biology when compared with features selected by differential expression analysis. We show how outliers can be used in conjunction with functional analysis to select interesting candidate genes, including novel tumour suppressors. Finally, in applying pathway analysis to outlier genes from the metastatic samples, we show how mCOPA can highlight molecular networks implicated in very small subsets of tumour samples, and even individual tumours. Such variations point to mechanisms that may underpin individual differences in tumours, and reveal specific elements of regulation and pathway perturbation.

mCOPA provides a new tool for the understanding of cancer heterogeneity and individual differences as captured in expression array experiments. Additionally, as most existing microarray studies have used differential expression analysis, the opportunity exists to use outlier tools such as this to reanalyze and reinterpret existing data with far greater granularity. Sample-specific analysis requires new ways of interpreting results, and the integrated methods we apply here demonstrate such new approaches. In combination with well-structured experimental design and clinical annotation, sample-specific analysis creates an opportunity to identify the mechanisms underlying rare disease subtypes and map these variations to individual differences in etiology and treatment response. mCOPA provides insight into the unique transcriptional landscape and molecular networks of individual patients or samples, and represents one of a new breed of bioinformatics tools designed to provide the analytical capability required for computational analysis in personalized medicine.

## Abbreviations

DE: Differential expression; COPA: Cancer outlier profile analysis; mCOPA: Modified cancer outlier profile analysis; ARI: Adjusted Rand index.

## Competing interests

The authors declare that they have no competing interests.

## Authors’ contributions

CW developed the algorithm and implemented the software. CW and MJD performed analysis on the Tomlins dataset, and interpreted the results. AT and SRM performed the comparative evaluation of clustering performance using public datasets. CCN and MAR provided guidance and resources for the project. CW, AT, SM and MJD contributed text to the manuscript. All authors read and approved the final draft.

## Supplementary Material

Additional file 1 **PublicDatasets.** Details of 12 datasets used in the evaluation. Click here for file

Additional file 2 **ARIScoresInEachExperiment.** Analysis of the significant differences of each method in each experimental dataset. Click here for file

Additional file 3 **ARIScoreDistributions.** Boxplots showing the distribution of ARI scores for each experiment. Click here for file

Additional file 4 **AnalysisOfFeatures.** Comparison of the features selected by each method. Click here for file

Additional file 5 **AnalysisOfGOterms.** Comparison of the GO terms selected by each method. Click here for file

Additional file 6 **TomlinsFeatures.** Outlier features identified in the Tomlins dataset. Click here for file

Additional file 7 **PathwayAnalysis.** Detailed outlier-based pathway analysis of metastatic samples from the Tomlins data. Click here for file
