# Supplementary figures and images for: mCOPA: analysis of heterogeneous features in cancer expression data
Source: J Clin Bioinforma. 2012 Dec 10;2:22. doi: 10.1186/2043-9113-2-22 (PMC3553066; doi:10.1186/2043-9113-2-22)

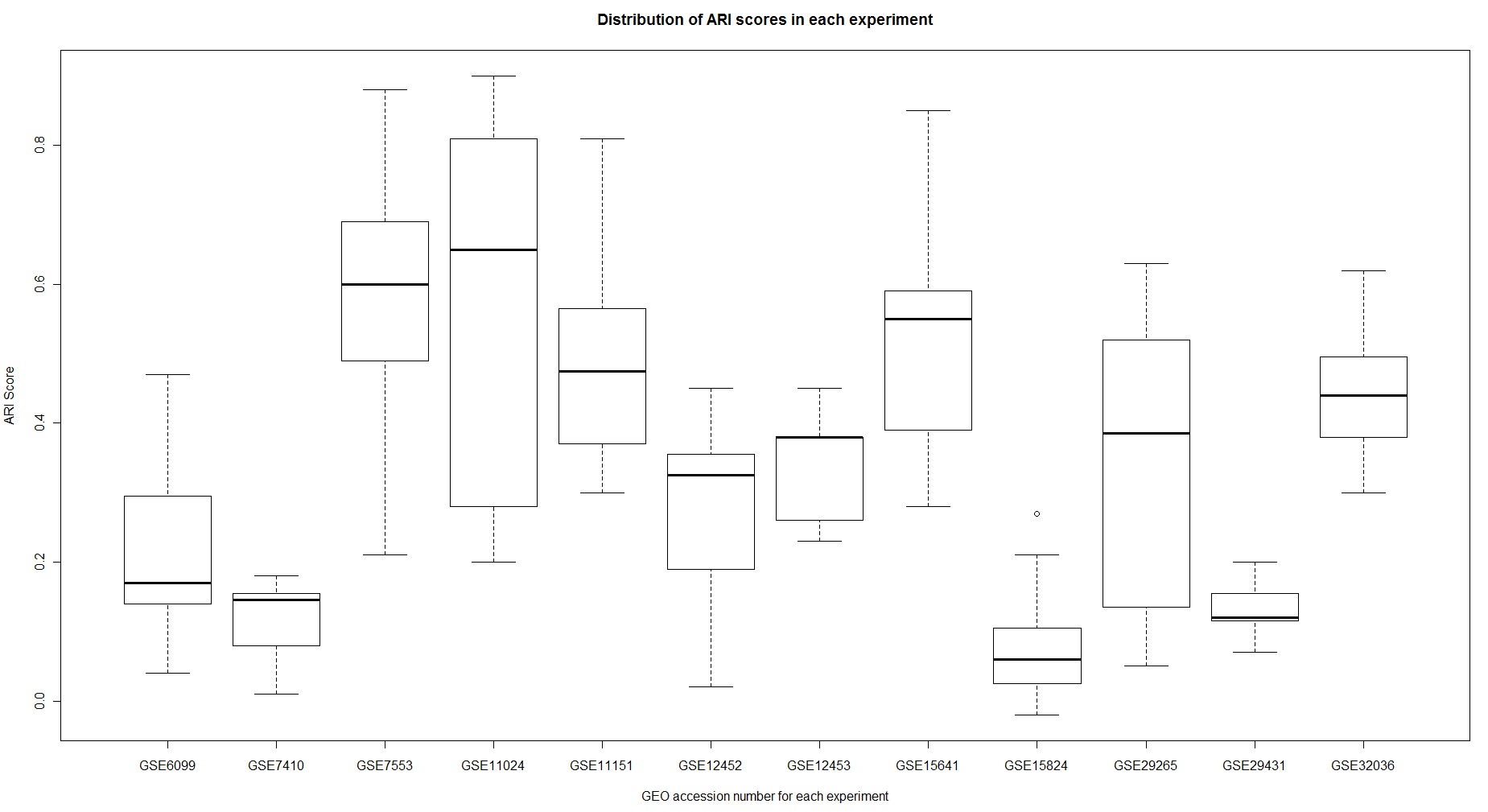

Supplement: Additional file 2 — ARIScoresInEachExperiment. Analysis of the significant differences of each method in each experimental dataset. [file 2043-9113-2-22-S2.jpeg]
